# Aligning with the 3Rs: alternative models for research into muscle development and inherited myopathies

**DOI:** 10.1186/s12917-024-04309-z

**Published:** 2024-10-18

**Authors:** Hashir Mehmood, Paul R. Kasher, Richard Barrett-Jolley, Gemma L. Walmsley

**Affiliations:** 1 Department of Musculoskeletal and Ageing Science, Institute of Life Course and Medical Sciences, Faculty of Health and Lifesciences, University of Liverpool, William Henry Duncan Building, 6 West Derby Street, Liverpool, L7 8TX UK; 2 Division of Neuroscience, School of Biological Sciences, Faculty of Biology, Medicine and Health, Manchester Academic Health Science Centre, The University of Manchester, Oxford Road, Manchester, M13 9PT UK; 3 Geoffrey Jefferson Brain Research Centre, Manchester Academic Health Science Centre, Northern Care Allianceand the, University of Manchester , Manchester, M6 8HD UK; 4 Department of Small Animal Clinical Sciences, Institute of Infection, Veterinary and Ecological Sciences, University of Liverpool, Leahurst Campus, South Wirral, Neston, CH64 7TE UK

**Keywords:** Muscle, Myopathy, Models, Replacement, 3Rs, Zebrafish

## Abstract

Inherited and acquired muscle diseases are an important cause of morbidity and mortality in human medical and veterinary patients. Researchers use models to study skeletal muscle development and pathology, improve our understanding of disease pathogenesis and explore new treatment options. Experiments on laboratory animals, including murine and canine models, have led to huge advances in congenital myopathy and muscular dystrophy research that have translated into clinical treatment trials in human patients with these debilitating and often fatal conditions. Whilst animal experimentation has enabled many significant and impactful discoveries that otherwise may not have been possible, we have an ethical and moral, and in many countries also a legal, obligation to consider alternatives. This review discusses the models available as alternatives to mammals for muscle development, biology and disease research with a focus on inherited myopathies. Cell culture models can be used to replace animals for some applications: traditional monolayer cultures (for example, using the immortalised C2C12 cell line) are accessible, tractable and inexpensive but developmentally limited to immature myotube stages; more recently, developments in tissue engineering have led to three-dimensional cultures with improved differentiation capabilities. Advances in computer modelling and an improved understanding of pathogenetic mechanisms are likely to herald new models and opportunities for replacement. Where this is not possible, a 3Rs approach advocates partial replacement with the use of less sentient animals (including invertebrates (such as worms *Caenorhabditis elegans* and fruit flies *Drosophila melanogaster*) and embryonic stages of small vertebrates such as the zebrafish *Danio rerio*) alongside refinement of experimental design and improved research practices to reduce the numbers of animals used and the severity of their experience. An understanding of the advantages and disadvantages of potential models is essential for researchers to determine which can best facilitate answering a specific scientific question. Applying 3Rs principles to research not only improves animal welfare but generates high-quality, reproducible and reliable data with translational relevance to human and animal patients.

## Introduction

“To move things is all that mankind can do, and for this the sole executant is a muscle, whether it be whispering a syllable or felling a forest.” This quote from Charles Sherrington describes the crucial role of the neuromuscular system in the biology of humans and other vertebrates. Skeletal muscles are architecturally complex tissues specialised to generate movement in response to stimulation; they account for 30—40% of total body mass in mammals (up to two-thirds in some species of fish) and consume much of the body’s energy demand. Contraction is actuated by acetylcholine release from peripheral motor neuron terminals at the neuromuscular junction, resultant opening of ligand-gated ion channels leads to depolarisation of the sarcolemma and a muscle action potential. Myofibres are highly developed to respond to this depolarisation by contracting in a timely, coordinated manner and thereby transmit force to the bones of the skeleton via the extracellular matrix [1, 2].

Disorders of muscle can be inherited or acquired, acute or chronic, focal or generalised and are an important cause of morbidity and mortality in human medical and veterinary patients [3, 4]. Clinical signs of myopathies can be variable and include debilitating muscular weakness leading to paresis of limb, postural and/or respiratory muscles; other symptoms may include exercise intolerance, fatigue, myalgia or muscle cramps and muscle atrophy, hypertrophy or contracture [4, 5]. Inherited myopathies, which will be the focus of this narrative review, comprise a large number of disparate genetic conditions that predominantly affect skeletal muscle structure, metabolism or ion channel functions [4, 6]. The prevalence of inherited myopathies, collectively affecting approximately 1 in 6,000 individuals worldwide, underscores their significant impact on global health and individual quality of life [7].

A knowledge of the biology of the tissue affected and a thorough understanding of disease pathogenesis are important foundations for developing effective treatments for any condition. All models used to study disease have advantages and limitations; however, an understanding of all available options, including computational and *in-vitro* models and less sentient animals than mammals (such as invertebrates or embryonic stages), will allow investigators to determine the best way to answer their particular research question. The aim of this review is to discuss the 3Rs principles and describe alternative models to replace or reduce the use of mammals for research into inherited myopathies and other muscle disorders.

### Inherited myopathies: muscular dystrophies and congenital myopathies

Duchenne muscular dystrophy (DMD), an X-linked dystrophinopathy, is the most widely known inherited myopathy and the most common lethal inherited disease in humans worldwide [8]. In addition, there are over 60 muscular dystrophies recognised in humans that are characterised by similar degenerative, non-inflammatory pathology, and many are attributed to genetic defects affecting structural proteins of the sarcolemma and extracellular matrix [4, 9]. Congenital myopathies are rare structural myopathies, typically presenting in children, with distinctive morphologic abnormalities within myofibres that allow classification based on pathological features [4, 10]. These disorders exhibit significant phenotypic heterogeneity, sometimes even within genotypes, varying in inheritance pattern, age of onset, clinical signs, muscle pathology and rate of progression; hence, molecular diagnosis is the gold standard [11, 12].

Inherited myopathies are also increasingly being recognised and genetically characterised in veterinary patients due to improvements in molecular testing and greater access to whole genome and exome sequencing. Dystrophin deficiency (analogous to DMD) has been reported in cats and numerous breeds of dog [13–18], and other muscular dystrophies with mutations affecting α2-laminin, sarcoglycans, collagen 6 and α-dystroglycan have also been described [15, 19–21]. Dogs are unique, as the process of artificial selection to produce such a variety of different breeds in our domestic dog population has led to particular breed-associated traits and disorders in these closed populations [22, 23]. Centronuclear myopathies (CNM) are rare congenital myopathies in humans; however, the mutation causing autosomal recessive HACD1-CNM is disseminated in the Labrador breed [24], and other recessive, X-linked and autosomal dominant forms have been reported in Great Danes, Labrador Retrievers and Border Collies respectively [25–27].

### Animal models for inherited myopathy research

The last decade has seen dramatic advances in our understanding of the pathogenesis of muscular dystrophies [8, 28, 29] and congenital myopathies [30, 31], and, whilst there is still no cure for these debilitating and fatal conditions, several promising therapeutic candidates for specific disorders have entered clinical trials [32–36] or been licensed for use in the USA [37, 38]. This work would not have been possible without the use of animal models, particularly mice and dogs [18, 39–41]. Mice are the most widely used model organism – as mammals, they share similarities in terms of anatomy, physiology and genetics to humans, and they have advantages of small size, short life span and rapid reproduction. The first murine muscular dystrophy model *mdx* was discovered over 40 years ago as a naturally occurring dystrophin mutation in a research colony of C57Bl6 mice [42]; however, there are now over 60 mutant and transgenic mouse models used for DMD research alone [43]. The phenotype in many murine myopathy models is relatively mild, and there is a well-recognised translational gap between rodent research and clinical trials in human patients, particularly in the context of gene therapy [41, 43, 44]. Canine models are more limited to naturally occurring diseases; however, there are now several muscular dystrophy and congenital myopathy-associated mutations characterised in dogs and kept in research colonies [45–49]. Research on these large mammalian models has shown they can closely replicate the clinical features, disease progression and severity levels of the same conditions as in humans [18, 25, 41, 45, 46]. Dogs share the longest common history with humans and are closer than mice to humans in size, weight and complexity of organ systems [14, 22, 47, 50]; therefore, results of therapeutic trials may be more translatable into human patients however, the experimental design should account for phenotypic variability [46, 51]. Dogs are, in many societies, beloved working companions and family pets; hence, their use in research triggers more ethical debate than other animals [52, 53].

Our scientific and medical knowledge and, consequently, human and animal health and well-being have undoubtedly benefited from the use of animals in research. Yet understandably, particularly concerning the use of sentient animals such as mammals, this remains a controversial and emotive topic; as researchers, we have an ethical obligation to consider the experience of any animals used in experiments and how to minimise harm and improve their welfare. The “3Rs” principles for humane experimental technique, an ethical framework for reducing animal use in research while improving laboratory animal welfare and quality of research [54], are accepted worldwide, and consideration is a moral imperative and required by legislation, grant awarding bodies and scientific journals in many countries [55]. There are, however, numerous other disadvantages to mammalian models in addition to the ethical considerations. Their use is tightly regulated—in the UK, the use of protected animals is regulated by the Home Office and requires licensing of breeding facilities, research premises, researchers and the projects themselves [56]. Maintaining and housing breeding colonies of rodents and other mammals, therefore, requires specialised facilities and is costly. Rodents and the majority of other mammals undergo gestation in utero which has obvious disadvantages for developmental studies in comparison with organisms whose embryonic stages can be directly accessed for interventions and observations. Additional considerations include exposure to animal allergens, which are common respiratory sensitisers. Traditional animal facilities are also costly in terms of environmental impact from energy usage and generation of waste materials [57].

### 3Rs: replacement, reduction and refinement

Some of the first legislation on animal welfare, including the UK’s 1876 Cruelty to Animals Act [58], regulated vivisection and this remains a complex and emotive subject. The Universities Federation for Animal Welfare was established in 1926 to work with academics and other stakeholders using a scientific approach to improve the welfare of animals used in research by addressing husbandry and experimental techniques. Their work was instrumental in bringing about many important improvements in laboratory animal care and management, anaesthesia and regulation of experiments, including proposing replacement, reduction and refinement as part of The Principles of Humane Experimental Technique [54]. Over the subsequent decades, the 3Rs have been internationally accepted as guiding principles and received support from the scientific community and public opinion; their consideration is enshrined in legislation in many countries throughout the globe and required for relevant grant applications and publications [59–62]. The NC3Rs, a UK-based scientific organisation established 20 years ago, have updated the original definitions of the 3Rs to align more closely with modern research practices (Table 1) [63].

**Table 1:** Definitions of the 3Rs (replacement, reduction and refinement) in animal research. Table adapted from [64] with original definitions by Russell and Burch [54]

	**Original**	**Basic**	**Updated**
Replacement	The substitution for conscious living higher animals of insentient material	Avoiding or replacing the use of animals in areas where they otherwise would have been used	Accelerating the development and use of predictive and robust models and tools, based on the latest science and technologies, to address important scientific questions without the use of animals
Reduction	Reduction in the number of animals used to obtain information of given amount and precision	Minimising the number of animals used consistent with scientific aims	Appropriately designed and analysed animal experiments that are robust and reproducible, and truly add to the knowledge base
Refinement	Any decrease in the severity of inhumane procedures applied to those animals, which still have to be used	Minimising the pain, suffering, distress or lasting harm that research animals might experience	Advancing research animal welfare by exploiting the latest in vivo technologies and by improving understanding of the impact of welfare on scientific outcomes

Replacement, the first of the “R”s and the focus of this article, as originally proposed by Russell and Burch, states “the substitution for conscious living higher animals of insentient material” [54], i.e. with regards to animal experimentation, if non-sentient alternatives are available then they should be used. This is the first “R” to address as it will have the biggest impact on welfare by fully replacing animals with human volunteers or cellular, mathematical, and computational models. This can remove the need for animal experimentation entirely. Where this is not possible, partial replacement considers preferentially using animals that are less sentient and, therefore, incapable of experiencing suffering. Which animals are considered sentient is another source of debate and controversy; however, the distinction is based on our current scientific knowledge, which is continually evolving based on new evidence (for example, decapods were recently included in the AW(S)A 2022) [65]. Precise definitions in legislation will vary however, in the UK, sentient species which are protected by A(SP)A (1986) include vertebrates and cephalopods such as octopuses and squid. The use of other invertebrates (such as flies and worms) and immature forms of fish and amphibians (prior to the development of independent feeding) would be considered partial replacement, as would utilising primary cells and tissues from animals after euthanasia.

Reduction ensures that the minimum number of animals is used in each study in line with the scientific aims. The experimental design should ensure sufficient power for reproducible and statistically meaningful findings and robust conclusions. A consideration for many myopathy models is phenotypic variability [41, 42, 46, 51, 66], therefore, this should be taken into account, and careful planning of measurements, cohort size and statistical analysis are crucial. Reduction may also include methods to maximise the useful information obtained per animal – for example, repeated blood samples, muscle biopsies or the use of live-imaging modalities such as magnetic resonance imaging in a longitudinal study may be preferable to culling animals at specific time points (balanced against the welfare implications of repeated use). Publication of research (including negative findings), collaborations and sharing of resources and data also reduces unnecessary replication. There are now several databases that facilitate the online sharing of “omics” data sets, including those managed such as EMBL’s European Bioinformatics Institute (EMBL-EBI) [67].

Refinements to reduce suffering and improve animal welfare can be made to all aspects of animal use, from their husbandry and housing conditions to their experience during experimental procedures. This is important not just from a welfare and ethical perspective but also can impact the validity, reliability and reproducibility of studies as pain and stress have measurable effects on behaviour and physiology that can affect scientific results yet may not be externally apparent, particularly in prey species.

Since their inception, these principles have provided a framework for ethical animal research and are now embedded in the policies of national and international bodies and organisations that regulate, fund, conduct and publish research. The 3Rs should be considered whenever animals are to be used for research in order to ensure best practice in terms of responsible animal use, welfare and scientific methodology. Furthermore, in order to maintain transparency, the reporting of studies involving animals should meet certain standards. The ARRIVE guidelines (Animal Research: Reporting of In Vivo Experiments) by NC3R provide a list of recommendations to improve animal reporting in order to maximise the quality, reliability, and reproducibility of research without unnecessary animal use in the future [68, 69].

In the context of muscle development and inherited myopathies, there are a number of alternative models which can replace and/or reduce the use of mammals for research. Here, we will discuss several of the most promising and widely used: cell culture models, computational modelling, invertebrates and embryonic zebrafish (compared in Table 2).

**Table 2:**
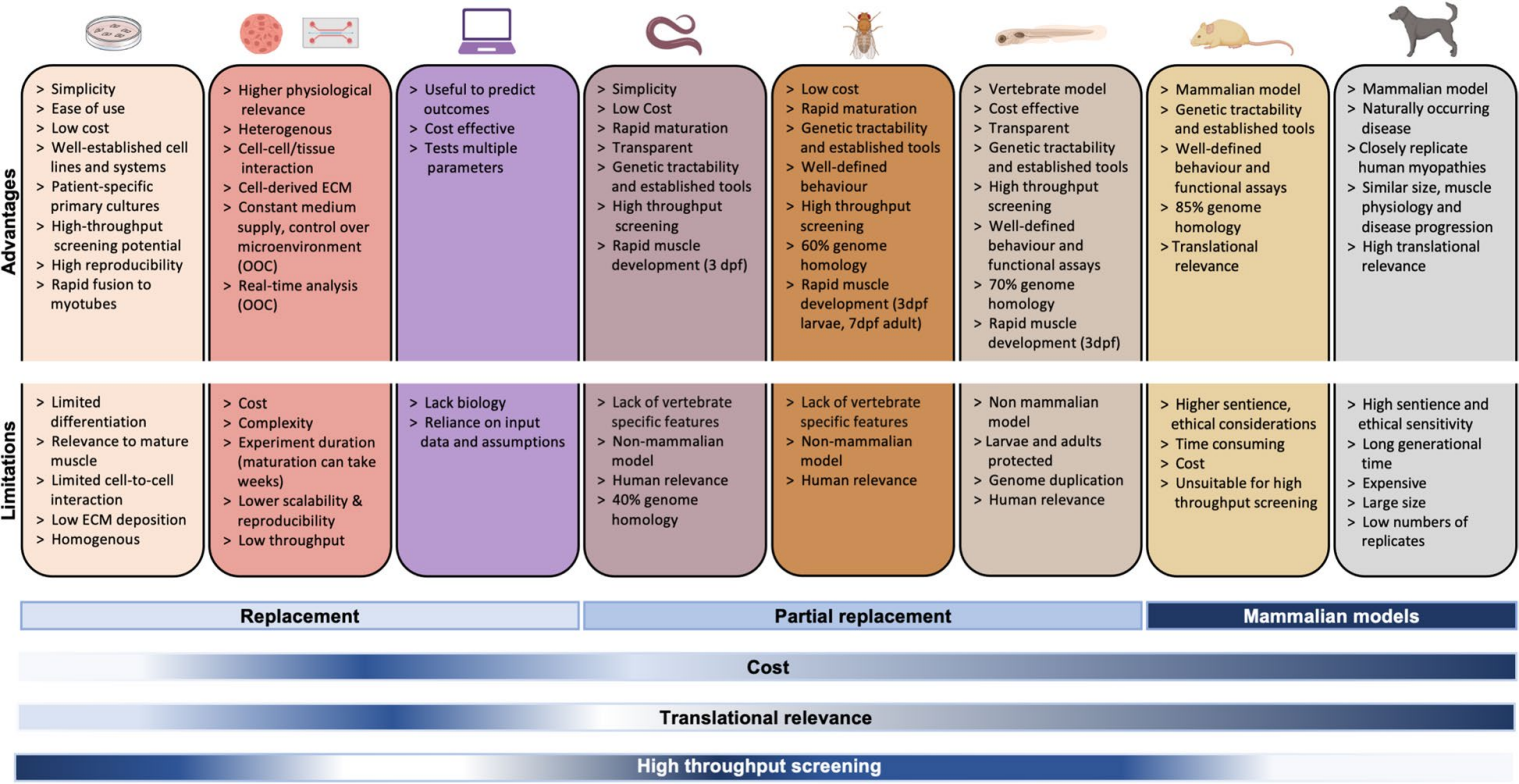
Comparison of alternative models for muscle development and inherited myopathy research. Created in BioRender.com

### Muscle in a dish – in vitro models

#### Cultured myoblasts

Cell culture models are commonly used to evaluate the development and pathophysiological mechanisms due to their low cost and ease of imaging and genetic manipulation; therefore, establishing and interpreting experiments in these isolated systems can be relatively inexpensive and straightforward. Established immortal myoblast cell lines from mice (C2C12) [70, 71], rats (L6) [72] and dogs (MyoK9) [73] are readily available and ethical replacements for animal models. Cell culture models also include immortalised and primary cells derived from the muscles of human patients [74] and animals; however, from a 3Rs perspective, the latter would be considered a partial replacement.

**Fig. 1:**
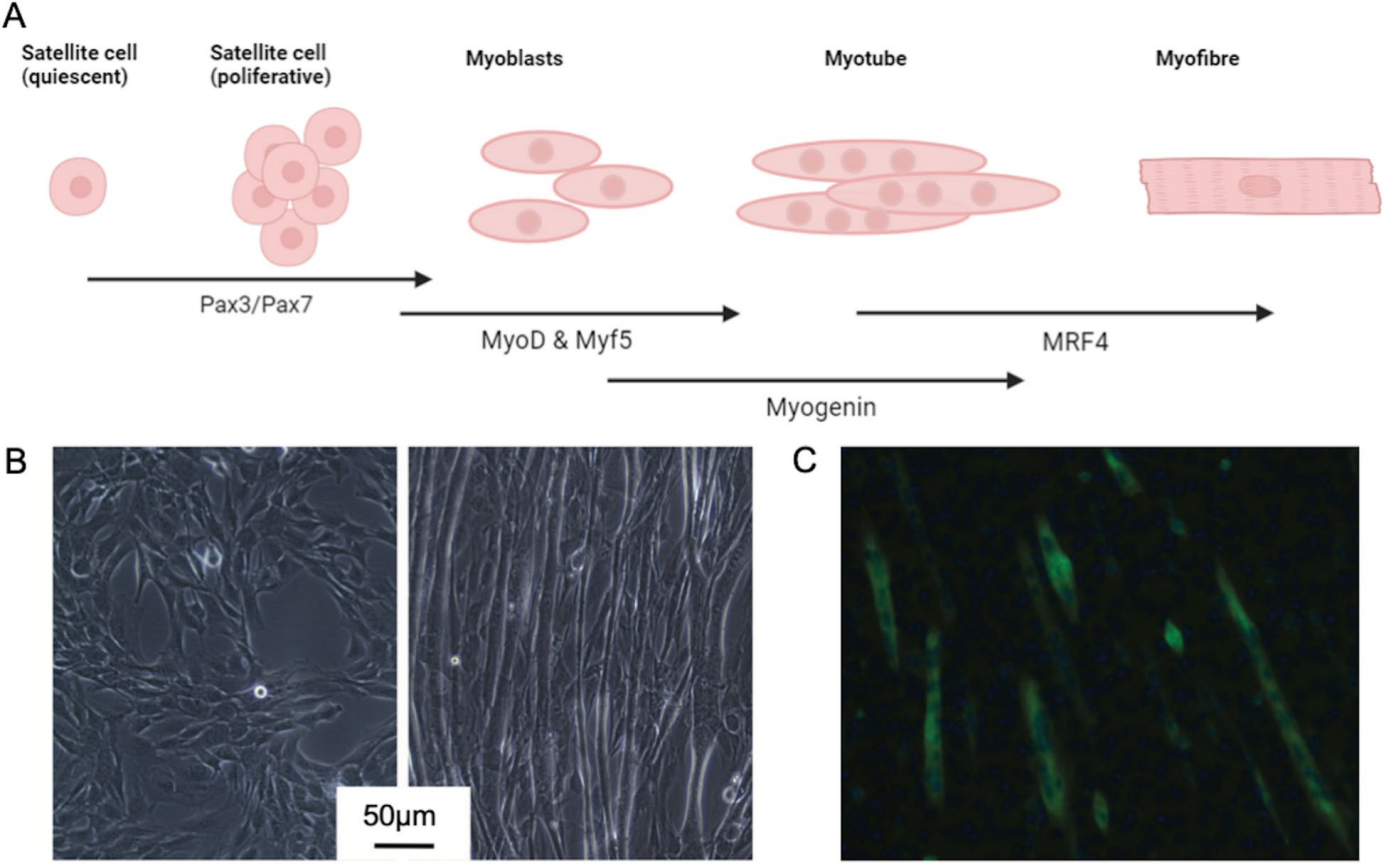
Myogenesis is a multi-step process regulated by a cascade of transcription factors. **A** Lineage specification of progenitors under the influence of Pax3/7 precedes expression of muscle regulatory factors (MyoD and Myf5), which commit cells and begin muscle differentiation by promoting expression of Mrf4 and Myogenin. Myoblasts, originating from the mesoderm, enter the cell cycle and proliferate. Upon withdrawal of growth factors, proliferating myoblasts exit the cell cycle and begin to differentiate: Myogenin and MRF4 are crucial in this step promoting elongation and fusion with neighbouring cells to form multinucleated myotubes. The myotubes begin to express muscle-specific proteins, such as cytoskeletal proteins (including sarcomeric myosin and α-actin), muscle creatine kinase (MCK) and ryanodine receptor 1. Mature muscle contains myofibres and a subset of progenitors forming the quiescent satellite cell niche, which can be activated for muscle regeneration. **B** Phase contrast images of C2C12 myoblasts (left) and myotubes differentiated to 7 days (right). **C** RYR1 immunocytochemistry with nuclear stain DAPI in C2C12 differentiated into myotubes at 8 days of serum starvation – note these are not organised as in mature myofibres. (Scale bar represents 50 μm). Created in BioRender.com

Primary myoblast cultures are formed from satellite stem cells within muscle samples, which become activated and proliferate [75]. These direct cultures retain many characteristics of the target tissue; however, they are relatively delicate cells, difficult to maintain and differentiate in culture, and they have a finite lifespan with limited capacity for proliferation; thus, they are impractical for studies requiring repeatable high-volume data [76, 77]. Conversely, myoblast cell lines that are immortalised (for example, by modifying telomerase activity or manipulating cell cycle checkpoints) can proliferate indefinitely, but genetic abnormalities can build up over time, leading to divergence of cellular behaviours from those observed in vivo [78, 79]. The murine C2C12 cell line [70, 71] is the most widely used of these and has been a cell culture workhorse for researchers of muscle development and biology for nearly four decades; they are easy to maintain, can rapidly and continuously proliferate in the correct conditions and then be induced to differentiate into myotubes for experiments as required (Fig. 1) [80].

Genetic manipulation is straightforward in cell culture (although transfection efficiencies are lower in myoblasts than some other cell types), and genetic material can be introduced into cells to cause transient or stable over-expression of mutant or tagged proteins, reduced expression or targeted genome editing [80–82]. For electrophysiological studies of channelopathies, including RyR1-related myopathies, cells without muscle-specific proteins can be advantageous, and HEK cells are commonly used [83]. Another strategy for disease modelling is to use cells taken from patients, thereby allowing researchers to study the disorder in its original genetic context and evaluate a range of mutations. However, human muscle biopsy samples are rare; therefore a platform for immortalisation of human myoblasts and MyoD transformation of fibroblasts has been established by the Institut de Myologie with over 130 disease models of 27 myopathies established to date alongside controls [74, 84]. Another potential source of human muscle cells is via induced pluripotent stem cells (hiPSC) [85, 86], which can be sourced from skin, blood and, more recently, urine [87]. This technology overcomes some of the disadvantages of patient muscle samples as they are highly proliferative and easily accessible.

The effectiveness of any in-vitro model relies on the origin and functional and structural maturity of its cells in comparison with the tissue being studied [88]. Skeletal myofibers are large, highly organised, multinucleate cells formed following a complex, multistep process controlled by sequential expression of myogenic regulatory and other transcription factors. First, proliferating myoblasts leave the cell cycle; they then elongate, migrate and align prior to fusion to form multinucleated myotubes and the assembly of striated myofibrils [89] (Fig. 1). Terminally differentiated muscle is challenging to study in vitro; however, myogenic cells grown in monolayer cultures can be readily differentiated into an intermediate myotube stage and have been used extensively to research early myogenesis and myopathies, leading to important insight into disease mechanisms for numerous conditions. For example, Blondelle et al., used Hacd1-deficient C2C12 to demonstrate altered lipid composition and impaired fusion as a cause of myofibre hypotrophy in CNM [90]. Genetically modified [82] or patient-derived primary and immortalised lines have been used to model several muscular dystrophies [74, 91–93] and in the field of personalised medicine, they have shown promise as a tool for disease modelling and drug screening: for example, evaluating the efficacy of antisense oligonucleotide exon-skipping and CRISPR/Cas9 genome editing strategies for DMD [94, 95]. Currently, iPSCs have been generated and validated for an array of muscular diseases, demonstrating their potential value for disease modelling and research [95–101]. Recently, micropatterned plates were used to establish primary myotube cultures in a high-throughput screening and drug discovery platform with quantitative phenotypic and functional outputs for myogenesis and hypertrophy/atrophy [102].

Such monolayer or 2D cell cultures are simple, inexpensive and user-friendly but their physiological relevance may be limited depending upon the research question being posed as they often fail to mimic the structural and functional complexity of mature muscles. Several methods have been described, including co-cultures, electrical or mechanical stimulation and 3D culture techniques, leading to improved myogenic differentiation in vitro models [103–106].

#### Three-dimensional cell culture

In recent years, significant advancements have been made in three-dimensional (3D) cell culture techniques, aiming to faithfully replicate the in-situ functioning of living tissues and overcome the limitations of 2D or monolayer cell culture. Common 3D cell cultures include 3D hydrogels, organoids, 3D bioprinting and organ-on-chips models and approaches are divided into scaffold-based and non-scaffold-based culture methods.

Scaffold-based methods involve introducing an artificial synthetic natural extracellular matrix (e.g., Matrigel, Collagen, Gelatin, Alginate, Polyglycol Acid, Polyethylene Glycol) to myoblasts to replicate the native mechanical environment [107]. Falcone et al., developed a 3D culture method for producing highly differentiated mature myotubes to evaluate myonuclear positioning in CNM—they were able to achieve sarcomeric patterning, t-tubules and peripherally located nuclei using primary murine myoblasts differentiated within a 3D gel of proteins replicating the extracellular matrix (Matrigel, BD Biosciences) during agrin treatment to stimulate the organisation of post-synaptic neuromuscular junctions [108]. Engineered culture substrates have also been beneficial in allowing improved differentiation and, therefore, modelling of patient-specific muscular dystrophy phenotypes and treatment responses [109].

Organoids are multicellular self-organised constructs derived from stem cells (including iPSC or ESC and primary tissues) that mimic the structure, cellular architecture and processes of the target organ [110]. Significant progress has been made in the creation of iPSC-induced muscle organoids [111–116]. For example, Mavrommatis et al. reported a gradual transition of early muscle progenitor cells (Pax3/7) to myotubes (MYH3) in a stepwise manner in iPSC-derived organoids [112]. Similarly, another study demonstrated hPSC-induced myogenesis and documented the presence of non-dividing satellite cells throughout differentiation that were later activated upon damage [113].

Further increasing complexity is organ-on-chip models (OOC), which combine 3D-engineered tissue within a microfluidic system to resemble in vivo organ-level physiology and pathophysiology. Unlike organoids, which form by random assembly of cells, the structure of OOCs is determined by deliberate planning of components such as biophysical and biochemical factors and cell numbers and types based on their physiological functions and relevance for disease [117, 118]. The tightly controlled microenvironment in OOC offers advantages for pharmacology studies and they permit high-resolution and real-time imaging, vascularisation, and diffusion of nutrients [119]. In the last few years, several studies have exploited microfluidic platforms to create muscle-on-a-chip models [120–122]. OOC technology can also allow us to study the interaction between several tissues or multiple organs, more closely modelling a whole animal [123, 124].

### Computational models

The use of in silico models to simulate and study complex biological systems has revolutionised life sciences research since the beginning of the twenty-first century. These have been driven by an almost exponential increase in computer processing speed. Storage space prices have dropped by equal measure, allowing the processing of vast datasets, including thousands of genes, simultaneously from thousands of cells [125]. Development of parallel processing (utilizing multi-core CPUs) and redeployment of graphical processing units (GPUs) with potentially thousands of processing cores, originally developed for the gaming industry, have also brought in the practical application of modelling techniques and machine learning models that were once theoretical, but now realistically achievable.

Such models now leverage mathematical and computational techniques to detect causal relationships, predict disease progression, treatment outcomes, and underlying interaction between different disease mechanisms and to identify potential biomarkers. Systems biology approaches aim to integrate information on protein interactions and molecular pathways up to whole organisms and their environment and predict their behaviour [126]. Predictive computation models have aided researchers in generating hypotheses and provide insight into cellular, pathogenetic and therapeutic mechanisms [127]. Since the BioModels repository was established in 2005 [128], there has been a vast expansion in the number of published and curated models available to researchers [129].

Several mathematical models have been developed to model mechanical aspects of skeletal muscle structure and contractile function in health [130–132] and also DMD, where a finite elements model was able to show which DMD-like changes in cell membrane and extracellular matrix properties lead to increased damage susceptibility [133]. Similarly, dynamical systems (ordinary differential equation-based) mathematical models have been applied to explore mdx mouse models of DMD [134, 135] explaining how the immune response can contribute to both muscle degeneration and regeneration. More recent, agent-based modelling is also a powerful simulation technique, where individual molecules are treated as individual “agents” with their own inherent rules and interactions [136]. As computer power grows, these have an increasing potential to model complex biological interactions [137]. For example, Virgillio et al. attempted to model many of the known pathogenetic mechanisms in DMD, including membrane fragility and altered inflammatory, fibroblast and satellite cell functions to predict the role of the microenvironment in the impaired regenerative response [138]. Using this modelling approach the authors were able to quantify the natural variability in muscle and establish parameters for potential treatment approaches aimed at cellular protection and functional preservation, which, paradoxically, can oppose each other.

Like all models, in silico models have their limitations; however, as they are based on known experimental results, and therefore, whilst they can incorporate many variables, the model is only as accurate and comprehensive as the data used. Developing these models also often involves some animal tissue data for early parameterisation. As this is an emerging field, there is limited research into the use of computational models for myopathy research specifically; however, the potential for computer models to replace animals in neuroscience research was recently reviewed [139] and found several areas from protein structure to behaviour where computational models performed well. The potential impact and utility of these models in the future are likely to grow with improved computer models and a further understanding of muscle biology and pathology.

### Invertebrates

#### Drosophila

The fruit fly, *Drosophila melanogaster*, has over a century-long history of use as a model organism in biological research and are well-established model for genetics and development, including muscle [140, 141]. Invertebrates such as *Drosophila* and *Caenorhabditis elegans* (covered below) represent some of the most simple and cost-effective model animals. They have numerous advantages, including a short life span, rapid sexual maturity (within 10 days for *Drosophila*), and the ability to produce a large number of progeny, allowing for large-scale experiments and fast progress [142]. The *Drosophila* genome is well-defined and relatively small (approximately 165 million bases encoding 14,000 genes versus around 3,400 million bases and 22,500 genes in humans) as there is less redundancy than in mammals – around 60% is homologous to the human genome, and 65–75% of disease-associated genes in humans have homologs in these flies [143–147].

Flies have multinucleate myofibers under neuronal control at the neuromuscular junction (NMJ) similar to that seen in mammals—although their neuronal circuitry is highly simplified, and the neurotransmitter at the fly NMJ is glutamate [148]. Their muscle development occurs in two phases: the first occurs during embryonic development, forming the body wall muscles needed for larval crawling (this musculature undergoes histolysis during metamorphosis), and the second stage of myogenesis generates muscles of the adult fly. Myoblasts forming both embryonic and adult muscles develop together during early embryogenesis. Whilst some leave the cell cycle to differentiate and create larval muscles, the adult muscle precursors continue proliferating and differentiate later during metamorphosis (pupal stages) [149, 150]. Both larval somatic muscles and adult-indirect flight muscles have been studied extensively, revealing similarities in both structure and core myogenic processes, including cell fusion and myofibrillar assembly, between mammalian and *Drosophila* muscles [151–154]. Myonuclei are positioned internally, unlike the peripherally located nuclei in mammalian muscle; however, nuclear positioning mechanisms appear to be somewhat conserved [155]. It was long thought that adult *Drosophila* muscles had limited regeneration capacity until the discovery of muscle stem cells relatively recently [156, 157].

The *Drosophila* genome has been sequenced and is well-annotated (the first assembly was published in 2020 [158]. A large, groundbreaking collaborative computational and experimental approach was used by the *Drosophila* research community to define functional elements and regulatory circuits [159] with a large repository of genetic and molecular data maintained at FlyBase [160]. There is an extensive and well-defined toolkit for genetic manipulation [146]. Early developmental studies used mutagenic screens, but the development of the *P*-element transformation vector allowed more precise manipulation and insertion of genetic material into the germline [161]. An extension of this, the GAL4/UAS expression system allows researchers to insert an RNAi construct or transgene into safe, well-characterised locations in the genome, allowing for gene silencing or expression respectively at specific developmental time points or in particular tissues [162]. Now, there is an array of readily available tools, including libraries of RNAi constructs/lines, large public stock. Repositories of mutant and transgenic strains (maintained by live culture and/or cryopreservation of ovaries or embryos) and reagents for targeted genome editing using ZFN, TALENs or CRISPR/Cas9 system [145, 146].

For many reasons, including ease of genetic manipulation, *Drosophila* has been used as human disease models, revealing several conserved pathogenic mechanisms and potential therapeutic approaches [163–165], and a growing body of new neuromuscular disease models are being established through these animals [145, 166, 167]. They exhibit simple, well-defined behaviours, which is an asset, particularly for neuromuscular disease research, allowing easy screening of their ability to crawl/climb and fly [166] alongside other functional assessments, including force-frequency relationship [168]. A variety of *Drosophila* muscular dystrophy models have been generated to model disease mechanisms and evaluate potential genetic disease modifiers and therapeutic strategies, including for DMD [169–172], oculopharyngeal muscular dystrophy [173, 174] and laminopathies [175] several of which were reviewed in detail by Plantie et al., [172]. More recently, fly models of myotonic dystrophies have been successfully developed that replicate key features of these diseases seen in humans and have contributed to our improved understanding of pathogenetic mechanisms and how the expanded repeats (in DMPK and ZNF9 in DM types 1 and 2, respectively) lead to many complex effects on various cellular processes via reduced gene function, RNA toxicity and altered splicing [166, 176–180]. Furthermore, therapeutic screens in these MD models have identified novel targets and potential therapeutic approaches that improve survival and locomotor activity [181, 182]. Drosophila has been extensively utilised as a model for sarcopenia and ageing research because of its accelerated development and short life span [167, 183, 184].

#### C. elegans

Another popular invertebrate model of muscle development is *Caenorhabditis elegans.* Like *Drosophila*, they offer several advantages, including fecundity, ease of use, low cost and absence of regulatory oversight. As worms, they are very simple animals and lack many key mammalian organs and physiological systems (including a heart and circulatory system and immune system). *C. elegans* has been well studied as a developmental and genetic model, and its genome was the first to be sequenced of any multicellular organism [185] as it is compact (100.3 Mbp containing around 20,000 genes); there are homologues for 60–80% of human protein-coding genes and around 40% of disease-causing genes [186].

*C. elegan*s muscle structure, general composition and cellular physiology have similarities to the skeletal musculature of vertebrates, and they have been used to model aspects of muscle development, particularly sarcomere assembly and maintenance [187, 188]. The (obliquely) striated body wall muscles used for locomotion extend longitudinally from head to tail and are composed of a single layer of rhomboid muscle cells [189, 190]. The function of these muscles can be evaluated by simple motility (thrashing) assays and other more sensitive assays are being developed [187, 191]. Thanks to their transparent cuticle, their muscle can be visualised in vivo*,* made easier with fluorescent protein reporter technology [189, 192]. There are, however, some notable differences: in particular, body wall muscle cells are post-mitotic, do not fuse during development and remain mononuclear [190, 193]. Ultrastructurally, the z-disc and costamere of vertebrate muscle are replaced by a dense body in C. elegans, which provides a similar anchoring function linking actin filaments to the extracellular matrix [189]. In addition, they lack satellite cells (muscle stem cells) required for regeneration/repair mechanisms; however, for some degenerative conditions, this can simplify interpretation and is seen as an asset [167].

The majority of *C. elegans* are self-fertilizing hermaphrodites that are able to produce large numbers of genetically identical offspring, which is ideal for large-scale experiments to evaluate therapeutic strategies and genetic interactions. Genetic manipulation is straightforward in vivo*,* and they are amenable to various well-described methods for genetic manipulation, many of which were developed in this organism [194, 195]. One advantage is the variety of methods to introduce genetic material, including adding E. coli containing engineered plasmids (e.g., expressing dsRNA for interference) to their diet [194]. Recently, genome editing techniques have become prominent in this organism, as for others discussed. Various tools are now established and available for researchers, including online databases and repositories of information (Wormbase) and libraries of mutant and transgenic worm cultures [195].

*C. elegans* is well-described as an invertebrate model of Duchenne muscular dystrophy [196]. Like DMD patients, dys-1 mutant worms (particularly the dys-1(eg33) mutant [197] have muscle degeneration and impaired motor function, which are exacerbated by concurrent mutations in hlh-1 (a homologue for myoD) [198] and improved by treatment with prednisone [199, 200]. More than 1000 compounds have been screened in this model with over 20 candidate drugs identified (summarised in Wasala et al., [201]; the translatability of these findings is unclear; however, glucocorticoids, hormone-related therapies (e.g. serotonin and melatonin) and cyclosporin A have been trialled in other models and human patients with varying success [196]. Recent work has revealed altered mitochondrial function as a novel target and potential therapeutic strategies to protect against this deterioration, including febuxostat [202], MA-5 [203] and modulation of sulfur metabolism by supplementation with hydrogen sulphide [199] or sulphur-containing amino acids [204]. *C. elegans* have orthologues for many genes important to muscle function in addition to the dystrophin-associated glycoprotein complex [196, 205, 206] and have been used to model aspects of several myopathies and neuromuscular disorders, including sarcopenia [145, 167, 207–209].

### Embryonic zebrafish

The embryonic zebrafish (*Danio rerio*) is another long-standing developmental model that has undergone a more recent resurgence in biomedical research [210]. As vertebrates, they have intermediate complexity between mammalian and invertebrate models, allowing researchers to harness advantageous features of both, including rapid, external development of large numbers of transparent embryos, ease of breeding, genetic manipulation and imaging, as well as the ability to model vertebrate-specific features and complex organ systems [211–213]. From a 3Rs perspective (in the UK and other European countries), zebrafish larvae are protected from five days post fertilisation (dpf) (when they have developed to allow independent feeding) [62, 214], and, whilst they are inexpensive to maintain in comparison with mammalian models, breeding adults are housed in licensed aquarium facilities. There is an estimated 70% homology between the human and zebrafish genomes, and more than 80% of human disease-causing genes have orthologous counterparts in zebrafish [215].

**Fig. 2:**
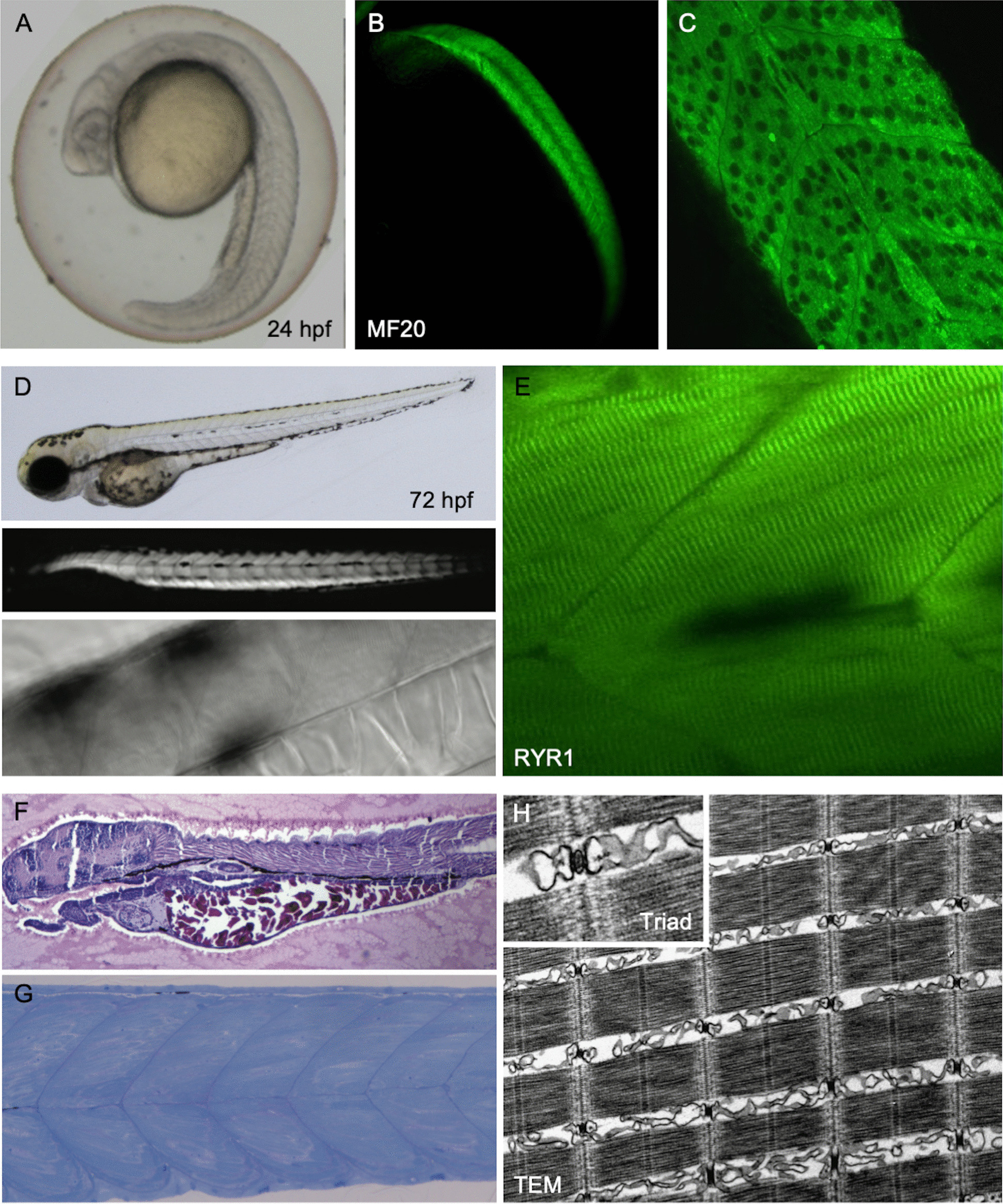
Muscle development in embryonic zebrafish at 24 (**A-C**) and 72 (**D-H**) hours post fertilization (hpf). Representative live AB WT embryos are shown in **A** and **D** (top), birefringence (**D**, middle) and differential interference contrast imaging (**D**, bottom) can also be used live to view muscle features including organisation and striations. Immunohistochemistry for sarcomeric myosin (MF20) **B**,**C** demonstrates myotubes in the somites (imaged using widefield fluorescence **B** and confocal *C* microscopy). **F** H&E stained gelatin-embedded cryosection and **G** toluidine blue stained semi-thin section of 72 hpf embryos. At this stage the myotomes contain mature myofibres with organized, striated multi-nucleate myofibres revealed by RYR1 immunohistochemistry **E** and electronmicroscopy **H**

Skeletal muscle is the largest and most prominent organ in developing zebrafish, sharing numerous developmental, molecular, histological, ultrastructural and pathological features with mammalian muscle [216, 217], including excitation–contraction coupling machinery, contractile apparatus and components of the dystrophin-glycoprotein complex [218–221]. Zebrafish embryos undergo rapid myogenesis: multinucleate myotubes develop within somites in the first 24 h corresponding with the onset of movement (spontaneous tail coiling), and mature myofibres are present within myotomes of free-swimming embryos by 3 dpf (Fig. 2). This pre-protected stage is commonly used for muscle development and myopathy research due to the presence of striated myofibres with highly organised tubuloreticular membrane systems. Disorganisation of the normal muscle structure can be easily screened using birefringence and functional assays, including the touch-evoked swimming response and spontaneous swimming behaviour, which can be automated in 96-well plates and provide quantitative data for large-scale studies [222]. More detailed studies using electrophysiological recordings and biomechanical measurements have also been used to characterise muscle abnormalities [223]. As with any model, there are disadvantages and differences in comparison with the target tissue, human muscle: skeletal muscles have similar cellular and subcellular structures and machinery, yet their gross anatomy and forces on the musculoskeletal system are very different. Whilst fish have appendicular and axial muscle groups, the former is much smaller than in mammals and locomotion is tail-driven with alternating contraction of myotomal muscle where fast- and slow-twitch fibres are topographically separate [224–226].

Another asset of the embryonic zebrafish model is the ease of genetic manipulation and an expanding array of strategies for generating transient or stable effects. Morpholinos are short, stable, RNA-binding oligonucleotides that can be injected into early embryos to produce a short-term reduction in the expression of a gene of interest by interfering with translation or splicing of mRNA or blocking miRNA activity for 3–5 days during embryonic development, allowing for rapid screening of effects [227]. Similarly, injection of DNA or RNA leads to transient over-expression for localisation of tagged variants and rescue experiments. Stable mutants and transgenic lines have been generated by chemical mutagenesis (e.g. ENU) [228], transposon-mediated BAC transgenesis [229] or targeted genome editing using CRISPR/Cas9 [230], ZFN [231] or TALENs [232]. The zebrafish genome is sequenced and well annotated [233], and thanks to the zebrafish mutation project [234], there are now many mutants and transgenic lines readily available to researchers, including those expressing fluorescent reporters [234]. A potential source of complexity when using zebrafish as a genetic disease model is an additional genome duplication that occurred in ancestral teleost fish around 500 million years ago [235]. Although many duplicates have been lost in the intervening millennia, some genes may have more than one ortholog for researchers to account for [236].

A variety of myopathies, including muscular dystrophies [218, 219, 237–239] and congenital myopathies [220, 221, 240], have been modelled in embryonic zebrafish with phenotypes and disease mechanisms that are translatable to mammals and humans. The first dystrophin mutant was identified during a forward genetic screen for myopathic phenotypes following ENU mutagenesis, and this strategy revealed several myopathy models identified based on locomotion and/or structural abnormalities [241, 242]. Since then, according to some estimates, 75 out of 121 known myopathy-associated genes have been modelled in zebrafish using a variety of strategies, including morpholinos and genome editing [243]. Disease pathways appear to be highly conserved, and in addition, several zebrafish models for muscular dystrophies, including DMD and Ullrich congenital muscular dystrophy, more closely recapitulate the severity of the pathology seen in humans than the analogous mouse model [244, 245]. In addition to disease modelling, zebrafish have been used to identify new disease mechanisms. For example, Dowling demonstrated increased basal oxidative stress in addition to defective excitation–contraction coupling in the pathogenesis of RYR1-related myopathies using relatively relaxed mutant zebrafish; this was supported by findings in cultured myotubes from patients and ameliorated by antioxidant treatment [246]. Furthermore, zebrafish provide us with the unique opportunity to efficiently validate and characterise novel variants discovered by next-generation sequencing in small families with rare myopathies [222]. Very recently, a zebrafish model of SPEG-related CNM has been developed using CRISPR-Cas9 genome editing following on from the discovery that bi-allelic variants in *SPEG* cause congenital myopathy [247]. Similarly, mutations in *CCDC78* were identified as the cause of a novel CNM with prominent internal nuclei and atypical cores in a small family and studied in embryonic zebrafish [248].

Embryonic zebrafish models are also excellent models for high throughput screening for genetic disease modifiers and compounds for potential therapeutic strategies [201, 249–251]. As previously discussed, invertebrate models and yeast are commonly used for large-scale molecular screens, but for studies of complex organ systems (like the neuromuscular system), embryonic zebrafish have a distinct advantage as they are able to more faithfully reproduce phenotypic characteristics leading to translatable results [252]. Conversely, for commonly used mammalian models such as the mouse, their reproductive capacities and their overall size and practicality limit their usefulness for large-scale screens; furthermore, due to their optical transparency, live imaging of muscle can be conducted non-invasively in zebrafish embryos, whereas muscle evaluation in a mouse model would require invasive biopsy procedures or culling of animals [172, 253]. Several large-scale unbiased screens have been carried out in zebrafish models of DMD and other muscular dystrophies to determine if any licensed drugs could be used, alongside other strategies, to modify disease—several potential therapeutic candidates, including phosphodiesterase and cyclophilin inhibitors and fluoxetine, were identified [252, 254, 255]. Drug screens in zebrafish models have also been employed for congenital myopathies, including MTM [256], notably identifying modulation of autophagy with PIK3C2B knockdown [257] and neuromuscular junction stimulation [258], of which the latter strategy has shown some efficacy in human clinical patients. Zebrafish have a longer life span than invertebrates; therefore, whilst they have been used for sarcopenia research, this is much less common [167].

## Conclusions

The ability to move towards things that sustain us and away from danger is of evolutionary importance and common to all animals – it is, therefore, unsurprising that many aspects of muscle development, anatomy and pathology are conserved and share similar features between species. Animals, in particular rodents, have been used for many years to model human muscle and myopathies, enabling a vast expansion in our understanding of muscle biology and disease mechanisms – yet many questions remain unanswered, very few strategies that have shown promising results in an experimental setting have translated into the clinic and the majority of inherited myopathies remain untreatable [43].

This is an exciting time for muscle disease researchers as scientific and technological advances are allowing us to utilise and develop real alternatives to replace mammalian models. In addition to the 3Rs considerations, many of these models have other advantages over traditional mammalian models, including reduced costs and space requirements, speed of development, access to immature stages, ease of imaging and genetic manipulation and reduced environmental impact. Cellular models are indispensable tools for investigating muscle disorders, and several recent developments (including the use of stem cell-derived cultures from human patients and progress in tissue engineering techniques) have allowed us to generate more physiologically relevant in vitro models for muscle research. In the future, improvements that include skeletal muscle in human “body on a chip” technologies and computational models have the biggest potential for full replacement of animals for muscle disease research [259].

Partial replacement with less sentient organisms offers another alternative when a whole animal model is required due to the complexities and interactions of skeletal muscle in vivo. For example, mature myofiber structure is often required to study congenital myopathies as a major pathogenetic mechanism is the disorganisation of tubuloreticular membrane systems and defective excitation–contraction coupling [31], which are challenging to replicate in cellular models. Of the models described here, embryonic zebrafish, as vertebrates, are phylogenetically closest to mammals, and they have successfully been used to model numerous inherited myopathies. Recent progress in MTM research provides an excellent example of how embryonic zebrafish can be used as pre-clinical models and for high throughput screening to elucidate novel mechanisms and potential therapeutic targets, thereby reducing and partially replacing mammals [220].

Scientific discoveries utilising the models described here have undoubtedly afforded valuable insights into biological processes, disease mechanisms and potential therapeutic strategies. The impact of these findings, however, depends on their ability to translate into clinically relevant results in human patients. Anatomical, physiological, and molecular differences between model and target organisms and the inherent variation in subjects and their environment in the real world compared with a controlled experimental setting, are amongst the various factors proposed that might contribute to the well described “translational gap” [260, 261]. A rational, integrative, collaborative approach combining exploratory and hypothesis-driven research strategies and verifying findings across different models, whilst leveraging the advantages of each system, is likely to increase confidence for efficacy prior to human trials. A stepwise approach, utilizing in vitro and small organism models for high-throughput screening and mechanistic studies, complemented by refined in vivo pre-clinical trials using smaller numbers in rodent or higher mammalian models aided by and informing computational models, aligns with 3Rs principles and contributes to the ongoing pursuit of effective therapies and deeper insights into muscle-related disorders.

In summary, as stated by George E.P. Box, “All models are wrong, but some are useful”. The pursuit of ideal models — simple, inexpensive, readily available, and accurate — remains a constant endeavour in biological or muscle research. Researchers investigating muscle biology and myopathies can harness a diverse array of models; a thorough understanding of the advantages and limitations of each will enable them to tailor their approach to best answer specific research questions while adhering to the principles of the 3Rs. Although animal models remain indispensable for certain aspects of myopathy research, the judicious use of alternatives, combined with refined experiments on small animal cohorts, ensures the humane generation of high-quality, translational data with relevance to both human and veterinary patients.

## Data Availability

No datasets were generated or analysed during the current study.

## Abbreviations

C. elegans: Caenorhabditis elegans
CNM: Centronuclear myopathy
DMD: Duchenne muscular dystrophy
Dpf: Days post fertilisation
EMC: Embryonic stem cell
FSHD: Facioscapulohumeral dystrophy
hiPSC: Human induced pluripotent stem cell
MTM: Myotubular myopathy
NMJ: Neuromuscular junction
OOC: Organ on chip
3D: Three dimensional
